# Preparing for Pregnancy in Women with Systemic Lupus Erythematosus—A Multidisciplinary Approach

**DOI:** 10.3390/medicina58101371

**Published:** 2022-09-29

**Authors:** Ioana Cristina Saulescu, Daniela Opris-Belinski, Andra Rodica Balanescu, Bogdan Pavel, Nicolae Gica, Anca Maria Panaitescu

**Affiliations:** 1 Department of Internal Medicine and Rheumatology, “Sfanta Maria” Hospital, 011172 Bucharest, Romania; 2 Department of Internal Medicine and Rheumatology, Carol Davila University of Medicine and Pharmacy, 050474 Bucharest, Romania; 3 Department of Functional Sciences, Carol Davila University of Medicine and Pharmacy, 050474 Bucharest, Romania; 4 Clinical Emergency Hospital of Plastic, Reconstructive Surgery and Burns, 010713 Bucharest, Romania; 5 Department of Obstetrics and Gynecology, Filantropia Clinical Hospital, 011171 Bucharest, Romania; 6 Filantropia Clinical Hospital, 011132 Bucharest, Romania

**Keywords:** systemic lupus erythmatosus, pregnancy, autoimmune disorders, preconception counselling, anti-Ro antibodies

## Abstract

**Highlights:**

**What are the main findings?**

- Unified, practical approach for preconception counselling of SLE women.

**What is the implication of the main finding?**

- What can be learned by the rheumatologist: the need for early referral for preconception counselling, risk stratification including from obstetrical point of view (preeclampsia (PE), anaesthetic evaluation), in vitro fertilisation, allowed medication during pregnancy (SLE-specific or non-specific), the need to evaluate for other autoimmune conditions includ-ing for thyroid disease.
- What can be learned by the obstetrician: how to assess activity and damage in SLE including scoring, autoantibody evaluation, and allowed vaccination from the SLE point of view.

**Abstract:**

Pregnancy is one of the most challenging processes the human body is exposed to: the healthy mother can carry to term a genetically different new-born, while her immune system adapts to tolerate this new status and avoids rejection. In autoimmune disorders, motherhood is even more challenging, with additional medical counselling, mother care, and foetus development checks being necessary. While the aspects of supplementary mother care and pregnancy progress tracking are associated with well-established medical procedures and protocols, counselling, be it pre- or post-conception, is still underestimated and scarcely applied. Indeed, over the past decades, medical counselling for this particular population has changed significantly, but from a healthcare’s provider point of view, more is required to ensure a smooth, controllable pregnancy evolution. One of the most frequent autoimmune diseases affecting young females during their fertile years is Systemic Lupus Erythematosus (SLE). Like other heterogenous diseases, it exposes the mother to severe, organ-threatening complications and unpredictable evolution. Both the disease and its treatment can significantly affect the mother’s willingness to engage in a potentially risky pregnancy, as well as the likeliness to carry it to term without any impairments. A good collaboration between the patient’s rheumatologist and obstetrician is therefore mandatory in order to: (a) allow the mother to make an informed decision on pursuing with the pregnancy; (b) ensure a perfect synchronization between pregnancy terms and treatment; and (c) avoid or minimize potential complications. The best approach to achieve these outcomes is pregnancy planning. Moreover, knowing one desired prerequisite for a successful pregnancy evolution in SLE mothers is a stable, inactive, quiescent disease for at least six months prior to conception, planning becomes more than a recommended procedure. One particular aspect that requires attention before conception is the treatment scheme applied before delivery as autoantibodies can influence significantly the course of pregnancy. In this view, future SLE mothers should ideally benefit from preconception counselling within their agreed care pathway. A multidisciplinary team including at least the rheumatologist and obstetrician should be employed throughout the pregnancy, to decide on the appropriate timing of conception and compatible medication with respect to disease activity, as well as to monitor organ involvement and foetus development progress.

## 1. Introduction

Motherhood has become a viable option for women with autoimmune disorders such as systemic lupus erythematosus (SLE), provided careful planning and timing are granted to the future mother.

**Figure 1:**
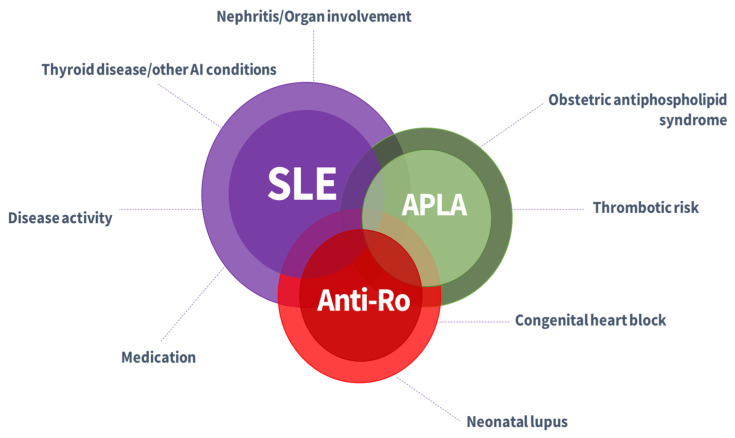
Pre-pregnancy aspects relevant for patients with known systemic lupus erythematosus. AI—autoimmune; SLE—Systemic Lupus Erythematosus; APLA—antiphospholipid antibodies.

The immune system protects the host against unwanted pathogens and eliminates them without hurting the host. In healthy future mothers, the immune function adapts smoothly and often effortlessly throughout the nine months of pregnancy. Associated autoimmune disease, particularly SLE, require a more complex perspective because of the different immunological set-up and associated medication. During an SLE pregnancy, there is extensive adaptation of mother’s immune response with the aim to allow safe development of the foetus and protect it [1]. Nidation will be a particular challenge since the embryo carries paternal genes and must not be rejected [2]. The foetus is genetically distinct from the mother, with half of the genes received from the father, pregnancy resembling an allograft that requires immune tolerance to be successfully carried to term [3,4]. Planned pregnancy is a must for women with SLE to avoid unfavourable outcomes. Preconception counselling enables the assessment of prognostic risk factors as well as the setup of a pregnancy plan according to patient’s medical history, health status, and medication scheme. Disease activity, medication plan, organs involvement, and autoantibodies should be carefully evaluated before pregnancy [5,6]. Figure 1 reflects relevant aspects to be considered in pregnancy planning for SLE women.

SLE is often encountered during childbearing age in women. In general, these patients have been shown to have smaller families [7,8], likely because of several interplaying factors such as: (1) disease activity or medications causing delays in pursuing pregnancy, (2) diminished ovarian reserve, (3) pregnancy complications with repeated failures and unfavourable outcomes, and (4) patients’, or even clinicians’, fear [8]. For these reasons, family planning should be addressed periodically as part of the treatment management of fertile SLE women.

A woman’s health at the time of conception is very important for a successful outcome [9,10]. Public health policies all over the world promote pre-pregnancy counselling to assess the mother’s health; to correct high-risk behaviours (smoking, alcohol, or illicit substance consumption); to perform screening for infections, including for various transmissible diseases such as HIV, Hepatitis, or TORCH (Toxoplasma Gondii, Other agents, Rubella, Cytomegalovirus, Herpes Simplex Virus); to recommend folic acid; or to check vaccination status [11,12]. Despite the increased awareness of the importance of pre-conception evaluation, real life brings a gap between knowledge and action. Healthcare providers, starting with general practitioners (GPs), obstetricians and gynaecologists or attending physicians should encourage women to plan and perform a preconception evaluation, whenever is the case. When a chronic disease such as SLE is present, this step is of utmost importance.

SLE is considered the prototype autoimmune disease with significant predominance in early adulthood females, mainly during childbearing age (15–45 years). It is also one of the most heterogenous disorders that can virtually affect any organ or system. Clinical manifestations can be very diverse, as the course of the disease can vary from longstanding quiescent to chronic remitting and relapsing. Morbidity is related to active disease but also to chronic damage accrual. With such an unpredictable evolution, pursuing normal desiderates such as a meaningful and accomplished social or family life could be very challenging, an aspect that clinicians should appropriately acknowledge when treating SLE women. Not long ago, SLE patients were advised not to pursue pregnancy because of fear of poor outcomes. This is not the case anymore. Nowadays, female SLE patients might have an uncomplicated pregnancy course with proper management and timing. However, these pregnancies still carry a higher risk than the those of general population females [13]. Patients and healthcare providers (regardless of specialty) should always collaborate in planning and analysing different disease scenarios, providing coherent and uniform information and preventing unwanted outcomes.

The aim of this review is to present relevant up to date information from a multidisciplinary perspective for adequate pre-conception counselling in SLE women and is intended not only for rheumatologists, but also for obstetricians, GPs, and anaesthesiologists.

## 2. Methods

We conducted a literature review of relevant scientific publications focusing on the impact of preconception evaluation and pregnancy in SLE female patients. Effects of SLE on fertility, pregnancy, and the new-born are discussed. Several medical databases, including PubMed, Google Scholar, and Cochrane Controlled trials Register, were used to access relevant information. The key words used for publication searching included: preconception, pregnancy, Systemic Lupus Erythematosus, and autoimmune disorder. The Journal Impact Factor and article citation score were used as the main citation criteria. We prioritized the latest guidelines, most cited reviews and meta-analyses, and most recent randomized controlled trials.

## 3. Results

### 3.1. Pre-Pregnancy Counselling from the Patient’s Perspective: When, Who, What, and Why

Formulating recommendations regarding family planning, women’s health, or medication during pregnancy and lactation has become possible in the recent decades after achieving a better understanding of the pathogenic mechanisms involved in SLE. Increased access to relevant data and experience-based treatment regimens aids the appropriate formulation of such recommendations. The first sets of guidelines on SLE and antiphospholipid syndrome (APS) were released by the European Alliance of Associations for Rheumatology (EULAR) in 2016, followed by the Canadian Rheumatology Association in 2018 and by the American College of Rheumatology in 2020 (ACR) [5,14,15]. All emphasise the importance of preconception counselling and militate for planned pregnancy in women with SLE. Pre-pregnancy counselling was recently evaluated from a patient’s perspective in a questionnaire-based study published in 2021 and conducted on a total of 124 SLE female patients. The majority of respondents mentioned that they would prefer to receive information about pregnancy from a healthcare provider (rheumatologist and/or gynaecologist) (81%) immediately after SLE diagnosis (53%) and together with their partner (69%). An interesting finding was that 16% of participants abandoned the desire to have children after pre-pregnancy counselling. This study highlights the need for women with SLE to be timely informed by a trusted physician with regard to any disease-related risks and complications during pregnancy in order to ensure an informed decision is made [16].

SLE is a chronic, female-predominant, lifetime disease that often starts before women engage in a pregnancy. Concerns about fertility issues related to the disease itself or associated medications are frequently raised by potential prospective mothers. Aspects such as disease activity or the need for medication during pregnancy, autoantibodies’ effect, and pregnancy complications with impact on either the mother or the child must be addressed during preconception counselling. Strong emphasis on the importance of the right time for a better outcome is mandatory [5,14,15,17].

As treating physicians, rheumatologists should consider fertility whenever a young female is diagnosed with and treated for SLE. A diminished ovarian reserve is often found in SLE patients, as demonstrated by low levels of anti-Mullerian hormone, making the fertile window for these women narrower [13,18]. So far, there is no evidence for any direct correlation between premature ovarian failure and disease activity or damage accrual, except for cyclophosphamide exposure [18,19]. Mycophenolate, azathioprine, calcineurin inhibitors, steroids, and the association of cyclophosphamide with gonadotropin-releasing hormone (GnRH) analogues appear to pose a lower risk when compared with the administration of cyclophosphamide alone. No evidence is available for this new biological treatment [18,19,20]. Effect of medication on fertility and available options for fertility preservation (oocyte preservation), should be part of the family planning discussion with SLE women considering a future pregnancy. Reassuring SLE patients that planning is necessary for a better outcome is expected to reduce the psychological distress.

### 3.2. Pre-Pregnancy Counselling from the Doctor’s Perspective: When Is the Right Time for a SLE Patient to Consider a Future Pregnancy?

#### 3.2.1. Disease Activity and Pregnancy Planning

SLE pregnancies are associated with higher risks and need more frequent follow-up checks with the obstetrician, rheumatologist, and family physician. SLE women that prospect motherhood should be aware of the possible disease flares during pregnancy and postpartum and of the pregnancy complications or unexpected outcomes for them or the child [20,21]. A cross-sectional analysis published in 2019 investigated trends in maternal and foetal complications among pregnant women with SLE. This retrospective study analysed data from United States between 1998 and 2015. The study looked for within-hospital maternal mortality, foetus mortality, PE or eclampsia, and non-delivery hospital admission. Overall, results showed a reduction in maternal mortality and unfortunate pregnancy outcomes in mothers with SLE, most likely as a consequence of improved disease-pregnancy timing. Similar results were found in other cohorts, too [22,23,24,25,26].

EULAR stresses the fact that pregnancy could be an option in the absence of active SLE. Disease activity or flares in the preceding 6–12 months before pregnancy are considered high risk factors for adverse maternal and perinatal outcomes [5,14]. For women with rheumatic and musculoskeletal diseases, including SLE, ACR guidelines also discourage planning a pregnancy unless quiescent/low disease activity is achieved [15]. These guidelines align with relevant evidence presented in several studies [27,28,29].

Alternatively, both the physicians and patients should be aware of possible complications related to an active disease status at or close to conception. Analysis of possible fortunate and unfortunate delivery outcomes, as outlined in Table 1, could potentially help women with SLE consciously decide on and plan a future pregnancy, including postponing the event until timing is more favourable. Additionally, assisted reproductive techniques are now available for SLE patients in many centres around the world and should be discussed in selected cases.

SLE is a heterogenous disease, with variable clinical manifestations and haematological, biochemical, or immune abnormalities characterised by an unpredictable evolution. Assessment of disease status should be based on clinical judgement and quantified using activity indices. Such an assessment should address both disease activity and severity, according to organ involvement and gravity of the dysfunction [33,34,35]. Rheumatologists are expected to provide a complete history of disease manifestation, symptoms, and a thorough physical exam, while the obstetricians and the GPs should always be able to recognise signs of active disease. Lupus rash, arthritis, and pain due to serositis are easy to detect and should prompt further medical evaluation in order to establish any correlation with an active disease. Some specific-organ involvement, such as renal or haematological involvement, might be clinically silent at the beginning, outlining the need for additional laboratory tests (urinalysis, proteinuria, complement level, specific autoantibodies, complete blood count). Although rheumatologists could use various tools to quantify disease activity before or even during pregnancy, some of these tools are scores mostly applied under research conditions and are therefore difficult to perform on a daily basis. Scores could also help assess disease activity in non-major organs, such as musculoskeletal or muco-cutaneous [35]. Systemic Lupus Erythematosus Disease Activity Index 2000 (SLEDAI-2K) is a validated, cumulative, and weighted index that allows the evaluation of the disease activity by the presence of any kind of the twenty-four different disease descriptors (neuro-psychiatric, visual, vasculitis, musculoskeletal, renal, cutaneous and mucous, serous, immunological, and haematological involvement) in the last 30 days and has the advantage of clear definitions. It is easy to apply, with online calculators available, and offers a relatively sharp perspective about the presence of active disease [35]. The score range is between 0 and 150. An episode of active disease is considered mild when the score is more than 4 but less than 6; moderate with a score of 7 to 12; and severe when more than 12 [36,37]. Moreover, the 2019 updated EULAR recommendations for treatment of SLE suggests treatment stratification according to these scores categories [37]. Although criticism around SLEDAI exists, this score has the advantage of being adapted for pregnancy, namely, the Systemic Lupus Erythematosus Pregnancy Disease Activity Index (SLEPDAI) [38], allowing for consistent evaluation when monitoring SLE pregnant women [36,38]. This instrument takes into consideration mimicry between different SLE manifestations and pregnancy, such as chloasma, oedema, headache, proteinuria, etc., helping clinicians to consider differential diagnosis when assessing these patients. The SLEPDAI might be helpful in monitoring pregnant women with SLE [38], making the SLEDAI an adequate pre-pregnancy choice.

SLE is a complex, systemic disease with a variable course hard to be captured in a single measurement. Defining activity, flares, remissions, and low-disease activity is still a matter of debate. Currently, there are no agreed standard cut-offs for the SLEDAI before conception, even if higher scores might mean higher risk for pregnancy complications. Usually, an SLEDAI-2K more than 4 means significant active disease [39], suggesting that a change in therapy might be necessary (adding immunosuppression, biologics, or addition or increase in corticosteroid dose) [37]. In such a context, associated risks if pregnancy will be pursued should be rediscussed at preconception counselling.

A validated definition for low disease activity, incorporating the SLEDAI, (where SLEDAI should be no higher than 4), is represented by the Lupus Low Disease Activity State (LLDAS). LLDAS takes into consideration the clinical evolution of disease, assesses changes since previous visits and monitors treatment (Prednisolone or equivalent no more than 7.5 mg daily and maintenance dose of immunosuppressive or approved biologics) [39]. 

Kim J.W. et al. published in 2021 a study showing that achievement of LLDAS will have a favourable impact on pregnancy outcomes. This retrospective study compared 163 SLE pregnancies with 596 pregnancies in the general population and confirmed that pregnant women with SLE will carry a higher risk of complications than those from the general population. Multivariate regression analysis positively corelated adverse outcomes with the lack of achievement of LLDAS before conception, suggesting that pregnancy should be postponed until LLDAS is achieved [40].

Along with activity, SLE evolution is associated with damage accrual, related to disease or to treatment. Specific conditions might contraindicate pregnancy due to high risk of maternal morbidity or mortality. End-stage organ damage such as heart failure, moderate to severe renal insufficiency (creatinine more than 2.8 mg/dL), severe pulmonary hypertension (estimated systolic pulmonary arterial pressure more than 50 mmHg or symptomatic) or pulmonary restriction, history of a stroke or major thrombosis in the last 2 years are such conditions [15,41,42,43,44,45].

Table 2 summaries the assessment measurements related to disease activity and damage in patients with SLE considering pregnancy.

#### 3.2.2. Treatment Regimens for Pre-Pregnancy and Pregnancy

Although a substantial risk exists, especially in SLE with specific organ involvement such as nephritis, pregnancy in SLE became possible after a better management of the disease with the improved available treatment. The advantage of a planned pregnancy is the adjustment of medication to control mother’s disease and foetus safety. Fertile women with SLE should always be informed about a treatment’s effect on a future pregnancy. SLE patients are often reluctant towards motherhood because of fear for disease activity and the need for medication during pregnancy. Informing them about the existence of pregnancy and breastfeeding-safe medication could alleviate such concerns. Moreover, advancements in foetal medicine and pregnancy ultrasound allow early detection of foetal abnormalities (12 weeks through pregnancy or later). A thorough specialised ultrasound scan at the end of the first trimester of pregnancy (first trimester scan, between 11 and 13 weeks) could provide reassurance to parents about the normal development of the foetus, excluding major malformations related to chromosomal abnormalities or teratogenic effects of drugs.

Table 3 emphasises the EULAR and ACR recommendations for specific SLE medication considered safe in preconception and during pregnancy. Acceptable medication should be continued if indicated to control active disease and prevent flares. When the patient is on an unacceptable medication, it should be changed to a pregnancy-compatible one, and its effects on the efficacy and tolerance should be observed before conception. Although no specific observation time was reported so far, a minimum of several months of observation is advised [5,14,15,46].

In addition to disease-specific medication, SLE patients might receive chronic treatment for hypertension or associated APS. Since some are contraindicated during pregnancy, switching to an approved class in preconception will allow for adapting treatment before pregnancy. In these situations, metoprolol and angiotensin converting enzyme inhibitors should be switched to methyldopa, nifedipine, or labetalol, where available, and oral anticoagulants to low molecular weight heparin (LMWH) [47,48].

SLE pregnancies carry a higher risk than general population ones for PE [49,50]. The 2019 National Institute for Health and Care Excellence (NICE) guidelines quantify autoimmune disorders as high risk for PE. Table 4 shows the classification of risk factors for high and moderate PE according to 2019 NICE guidelines [50]. The presence of one high risk factor or at least two moderate risk factors highlight that the women should be considered for prophylaxis with low-dose aspirin (LDA) [51]. EULAR recommends LDA in SLE pregnant women especially if they have a history of lupus nephritis or are positive for APLA (5). This indication is supported by the results of a multicentre study published in 2022 that evaluated the impact of LDA on pregnancy outcomes in SLE women [52]. ACR suggests that treatment with LDA should be considered for all pregnant women with SLE [15]. However, gaps between real life context and current recommendations might still exist: a study by Mendel A. et al. including 475 SLE pregnancies with risk factors for PE showed that half of them had additional risk factors (other than SLE), but LDA corresponded to only one-quarter. Moreover, the majority of patients with lupus nephritis and positive APLA did not initiate LDA in early pregnancy [49]. The same results were shown by Haase et al. [53]. Pre-conception evaluation should analyse supplementary risk factors for PE since this is a major contributor to the mother’s and foetus’ adverse outcomes. A careful individual risk–benefit balance is necessary to allow more women with SLE, especially those with additional risk profiles, to start LDA at pre-conception or early in pregnancy, improving the outcomes.

Low levels of Vitamin D are expected in SLE women since they are advised to use ultraviolet (UV) protection. According to EULAR, adjunct therapies with supplements such as calcium and Vitamin D should follow general population recommendations. Measuring 25-hydroxy-Vitamin D [25(OH)D] levels is usually performed only after the pregnancy is confirmed [5]. Pre-conception evaluation should initiate supplementation with vitamin D and calcium if the patient is not already on them, with a special attention to the presence of additional risk factors for low levels of vitamin D such as glucocorticoids or heparin treatment, malabsorption, and renal or hepatic insufficiency. There is no consensus about the daily dose of vitamin D. The Institute of Medicine of the National Academy (IOM) established in 2010 that an intake of 600 (400–800) International Unit (UI) per day for vitamin D and 1000 mg per day for calcium are adequate [54]. These recommendations target a 25(OH)D level of minimum 20 ng/mL [54], but more recent data demonstrated a pleiotropic effect for Vitamin D with a minimum level of 30 ng/mL of serum 25(OH)D at a vitamin D dietary intake up to 2000 UI daily [55]. We propose that at pre-conception evaluation, women with SLE with supplementary risk factors as already mentioned be tested for their levels of 25 (OH)D and that a higher regimen supplementation be addressed if needed. Folic acid administration should follow the general population indication and a dose of 0.4 to 0.8 mg per day should be started pre-pregnancy [56].

Patients presenting SLE have a low ovarian reserve, therefore complicating conception [57]. In vitro fertilization is a potential solution. In a study performed on 37 patients presenting SLE or APS, it was proven that this technique is safe, and 70% of them delivered at least one child [58]. A factor for this success could be represented by the treatment of patients with SLE with hydroxychloroquine, which is suspected to have a protective effect on chronic inflammatory lesions of the placenta [59].

#### 3.2.3. Autoantibodies and Pregnancy Complications and Foetal/Neonatal Abnormalities

The importance of APLA for SLE is highlighted by their inclusion in all classification criteria elaborated for this disorder [60,61,62]. The presence of lupus anticoagulant (LA), anti-cardiolipin antibodies (aCL), and anti β2 glicoproteina 1 antibodies (aβ2GP1) must be determined at diagnosis, but when planning a pregnancy, they should be re-evaluated. Persistent moderate–high APLA titres, LA, and multiple APLA positivity represent a high-risk APLA profile and are considered strong predictors for adverse maternal and foetal outcomes [5,63]. Association of SLE with APS-past pregnancy morbidities or thrombotic events [64]—will further increase the risk for future pregnancy morbidity and thrombotic events or PE, intrauterine growth restriction, death or preterm delivery, as shown in Table 5 [14]. In this situation, pre-conception counselling has the role to discuss with the SLE patient the added risk and establish a valid treatment plan: LDA for APLA high-risk profile, LDA and prophylactic LMHW for obstetric APS, and LDA and therapeutic LMHW for thrombotic APS [5,15].

**Figure 2:**
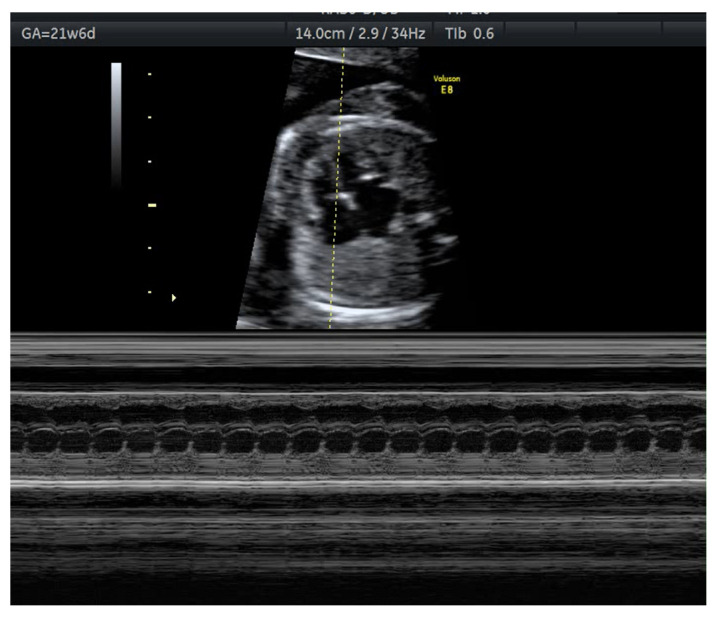
Foetal ultrasound M mode-foetal complete heart block in a pregnancy of 21 weeks of a mother with anti-Ro antibodies.

Anti-Ro/SSA and anti-La/SSB antibodies are also important for the preconception evaluation. They are linked to neonatal lupus and congenital heart block, especially in the presence of moderate–high titres of anti-Ro [5,14,65]. Increasing exposure to hydroxychloroquine during pregnancy and more frequent obstetric evaluation might contribute to a less stressful pregnancy. Figure 2 presents a case of complete atrio-ventricular block in a foetus of a mother with positive anti-Ro antibodies at 21 weeks of pregnancy. Unfortunately, there are limited options for the treatment of congenital block, beta-sympathomimetics, intravenous immunoglobulin, or apheresis being the main ones [66,67].

Thyroid disease is, in many clinical settings, evaluated in any woman that wants to conceive. Association of autoimmune thyroiditis and SLE is very frequent, implying the need for antithyroid antibodies testing before pregnancy. Women with hypothyroidism and anti-Ro antibodies have an increased risk for delivering a child with congenital complete heart block when compared with women with antibodies alone [68]. Evidence correlating preterm delivery with thyroid disease in SLE women was previously reported [69].

Maternal and foetal outcomes when exposed to different autoantibodies are summarised in Table 5.

#### 3.2.4. Vaccination

The recent SARS-COV-2 crisis has demonstrated that vaccination is one of the most important contributions of medicine to public health. Since one of the most important, yet vulnerable periods in the life of a woman is represented by the nine months of pregnancy, timely immunizations are mandatory. When SLE history is included in this context, concerns arise due to fear for precipitating the infection in a patient with abnormal immune response (when live vaccines are used) or stimulating the autoimmunity and starting a flare (with any vaccines). EULAR recommendations updated in 2019 for vaccinations in adult patients with autoimmune disease, clearly state the types of vaccines relevant in SLE and their relationship with SLE medication schemes. Rheumatologists should assess the vaccinations status and the need for further immunizations yearly in these patients. A shared physician–patient decision might have a significant contribution to the reduction in psychological distress related to this topic [70,71]. Moreover, EULAR stated in November 2021 that patients with autoimmune diseases (including SLE) should be advised to receive SARS-COV-2 vaccination with approved vaccines [72].

Vaccination status should be checked at preconception counselling and missing recommended vaccines updated before conception (influenza, SARS-COV-2, rubella, etc.).

#### 3.2.5. The Surveillance Team and Care Pathway

Desire for pregnancy and pregnancy planning should be discussed with SLE childbearing women from the moment of diagnosis. Rheumatologists should reassess these issues periodically, engaging patients as active partners on the path of a successful pregnancy, when they desire this. Patients’ knowledge about proper timing for conception, the need for tailored treatment, and the importance of pre-screening for autoantibodies and comorbidities will encourage contraception until conception is permitted [15,21].

**Figure 3:**
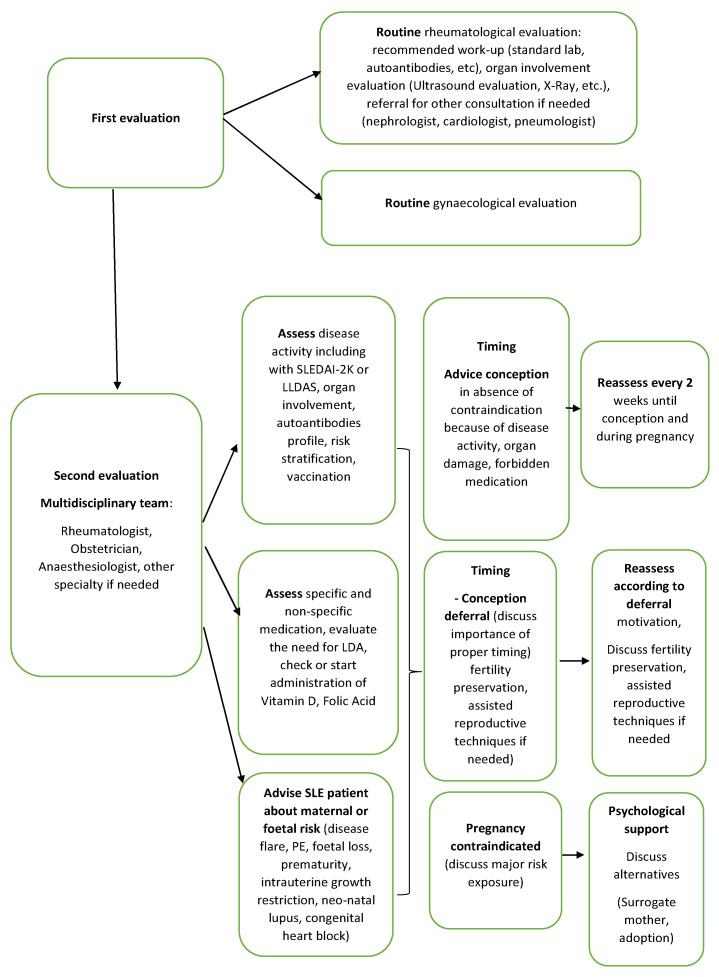
Multidisciplinary team approach for SLE women who desire to conceive. LDA—Low-Dose Aspirin; LLDAS—Lupus Low Disease Activity State; PE—Pre-Eclampsia; SLE—Systemic Lupus Erythematosus; SLEDAI-2K—Systemic Lupus Erythematosus Disease Activity Index 2000.

The main tools available for rheumatologists treating SLE women interested in pursuing a pregnancy are EULAR and ACR guidelines. “Healthy Outcomes in Pregnancy with SLE Through Education of Providers (HOP-STEP)” (www.lupuspregnancy.org, accessed on 10 June 2022) is a curriculum designed to guide rheumatology clinicians, helping them to accumulate necessary knowledge and confidence to guide successful pregnancy planning and optimise the outcomes [73]. Once the rheumatologist considers that disease status permits pursuing a pregnancy, a pre-conception counselling and evaluation must be the next step. Pre-pregnancy counselling has a favourable impact on both maternal and foetal outcomes, as already outlined in this review. As shown in Figure 3, a multidisciplinary clinical pathway for SLE women who desire to conceive is shown to reduce disease-related flares during pregnancy compared with pre-conception counselling only [48]. In this view, a team formed by the obstetrician, rheumatologist, and, on indication, nephrologist, cardiologist, pneumologist, and anaesthesiologist allow a thorough health check of the SLE mother as well as a good assessment of appropriate timing, medication, comorbidities, or vaccinations that are in line with approved recommendations. Secondary/tertiary healthcare facilities with expertise in attending SLE patients and SLE pregnancies is advised. Follow-up evaluation should be completed every two weeks, preferable in a multidisciplinary meeting until conception and during pregnancy [48]. Such a setup enables a correct differential diagnosis between SLE flares during pregnancy, pregnancy complications (lupus nephritis versus PE), and foetal complications related to autoantibodies. The aim of such an approach is to rapidly and correctly treat the patient experiencing complications, avoiding unnecessary medication or wasted time. Proposed pre-conception evaluations in a clinical setting are synthetized in Table 6 and Table 7.

#### 3.2.6. Anaesthetic Considerations

Knowing that many pregnant women need different surgical interventions during pregnancy, as well as Caesarean section (C-section) for the new-born’s delivery, the anaesthesiologist should also be aware of potential complications related to SLE. Consequently, the future SLE mother should be counselled about the anaesthetic options for delivery. In the case of a general anaesthesia, several drugs used to treat SLE can interfere with the anaesthetics. Cyclophosphamide has an inhibitory effect on pseudocholinesterase, and azathioprine interacts with muscle relaxants [74].

The hematologic changes presented in some SLE patients, such as thrombocytopenia and APS, which also require anticoagulation, could interfere (contraindicate) with regional anaesthetic techniques such as epidural for analgesia or combined spinal and epidural for C-section [75]. Fortunately, such hematologic changes are not markedly frequent, and regional anaesthetic techniques can be performed safely, but only after the assessment of blood coagulation tests and platelets count [76].

Cardiovascular tests must include 12 lead electrocardiogram and echocardiography for the exclusion of any signs of ischemia, pericarditis, or endocarditis. Pulmonary function assessment requires pulmonary function tests, chest X-ray (pleuritis, pleural effusion or interstitial disease), and arterial blood gas tests.

Due to the changes that can affect intubation, such as mucosal ulceration, temporomandibular joint malfunction, laryngeal dysfunction, and atlantoaxial subluxation, the pregnant woman should be advised regarding the possibility of using an awake fibre-optic intubation [77,78].

Patients presenting SLE are also prone to infection, so antibiotic prophylaxis must be taken into consideration.

## 4. Discussion

Over the past decades, substantial changes have been observed in the quality of life, survival rates, and pregnancy of SLE women. A more standardised evaluation, awareness of family planning’s importance, stratification of risks according to disease activity and comorbidities, and tailored medicine all contributed to the present paradigm of being pregnant with SLE.

Non-planned pregnancies are a common fact all over the world, but for SLE women, not choosing the right moment for conceiving might have maternal or foetal consequences. Fear experienced by both the treating physician and the patient in relation with a possible flare or complication during a future pregnancy will increase the likelihood of ambivalent patient in relation with pregnancy. Recent studies showed that most frequent non-planned pregnancies occur in SLE women that are not actively engaged in family planning or contraception during active disease or when using teratogenic medication [26].

This review stresses the importance of family planning starting the moment when a woman at a child-bearing age is diagnosed with SLE. Focusing on achieving quiescent disease with permitted treatment before conceiving must be the rule acknowledged by the team formed of doctors and treated patients [5,15].

Pre-conception evaluation performed in a multidisciplinary clinical pathway, with obstetricians, rheumatologists, and other specialists as needed and with the dedicated help of the general practitioner will contribute to an improved outcome, both for the mother and the child [48].

## 5. Conclusions

With particular interest in SLE patients, reproductive issues should be addressed by the treating physicians from the moment of diagnosis and as often as needed. Regarding pregnancy, preconception counselling and risk stratification are essential for the best outcome. The future mother’s knowledge about disease, medications, vaccinations, and comorbidities will help her to actively participate in the planning of pregnancy, helping her to understand timing, risks or possible evolution, therefore improving the pregnancy’s evolution and delivery.

## Figures and Tables

**Table 1:** Prognostic implications to be discussed at a preconception counselling.

Risk Factor	Increased Risk	References
SLE activity in the last 6–12 months or at conception	Flare during pregnancy Hypertension Foetal morbidity and mortality Preterm delivery Pregnancy loss	[5,30,31]
Lupus nephritis (anytime)	Renal flare during pregnancy Foetal loss Preterm delivery PE	[5,32]

PE—Pre-eclampsia; SLE—Systemic Lupus Erythematosus.

**Table 2:** Pre-conception counselling evaluation regarding disease status.

Evaluation for Preconception Counselling
Clinical evaluation: anamnesis (important to find out if patient needed to increase or to add Prednisone or immunosuppressive therapy in the preceding 6 months for active disease) and complete physical exam
Lab evaluation: complete blood count, proteinuria, urinalysis, renal and hepatic function, glucose level, coagulation tests, inflammatory markers
Immune marker associated with active disease: low complement (C) level (C3, C4, C1q) and increased anti-double-strand DNA (anti-dsDNA)
Assessment of organ involvement with specific investigation (ultrasound, pulmonary function, imaging if required) and activity scores (e.g., SLEDAI-2K, LLDAS)
Check for existence of damage that might contraindicate pregnancy: pulmonary hypertension, low pulmonary function, cardiac failure, severe kidney failure, stroke or major thrombosis

Anti-dsDNA—anti-double strand DNA; C-complement; LLDAS—Lupus Low Disease Activity State; SLEDAI-2K—Systemic Lupus Erythematosus Diseases Activity Index 2000.

**Table 3:** How to use specific available medication during preconception period.

Medication	Pre-Conception	References
Hydroxychloroquine	Recommended to all patients	[5,15,46]
Oral glucocorticoids	Accepted if needed at lowest effective dose, but less than 20 mg/day equivalent Prednisone	[5,15,46]
Azathioprine	Accepted if needed	[5,15,46]
Cyclosporin A and tacrolimus	Accepted if needed, caution if high blood pressure	[5,15,46]
Nonsteroidal antiinflamatory drugs	Accepted if needed, discontinue if there is problem with conceiving, cyclooxygenase 2 not indicated	[5,15,46]
Methotrexate	Stop 1–3 months prior to conception	[5,15,46]
Leflunomide	Stop if planning a pregnancy, washout with cholestyramine until no longer detected	[5,15,46]
Mycophenolate Mofetil and Mycophenolic Acid	Stop at least 6 weeks before conception to observe flare after discontinuation	[5,15,46]
Cyclophosphamide	Stop 3 months prior to conception	[5,15,46]
Available biologics (Belimumab, Rituximab)	Discontinue at conception	[15]
Recently approved biologic (Anifrolumab)	Not yet included in EULAR and ACR recommendations, no available data related to safety during pregnancy	

EULAR—European Alliance of Associations for Rheumatology; ACR—American College of Rheumatology.

**Table 4:** NICE stratification of risk factor for PE [50].

High Risk for PE	Moderate Risk for PE
History of hypertension disease in a previous pregnancy	Nulliparous
Maternal disease: chronic kidney disease, autoimmune disease, diabetes, chronic hypertension	≥40 years of age
	Body mass index (BMI) ≥ 35 kg/m^2^
	Family history of PE
	Multifetal pregnancy
	Pregnancy interval of more than 10 years

BMI—Body Mass Index; NICE—National Institute for Health and Care Excellence; PE—Pre-eclampsia.

**Table 5:** Association between different autoantibodies and maternal and foetal outcomes in SLE pregnancies.

Autoantibodies Profile	Maternal Outcome	Foetal Outcome	References
High-risk APLA profile: persistent moderate or high APLA titres, LA, multiple APLA positivity	Maternal vascular thrombotic events, PE	APS related pregnancy morbidity (unexplained spontaneous pregnancy losses before 10 weeks of gestation, preterm delivery before 34 weeks of gestation because placental insufficiency, unexplained foetal death after 10 weeks of gestation) Intrauterine growth restriction, Pre-term birth	[5,21,31,50,64]
Anti-Ro/SSA and anti-La/SSB antibodies		Congenital heart block Lupus neonatal	[5,15,21,68]
Antithyroid antibodies: tyroid peroxidase antibody tyroglobulin antibody tyroid-stimulating immunoglobulin antibody tyroid-stimulating hormone receptor binding inhibitor immunoglobulin		Preterm delivery Increased risk of complete congenital heart block in association with anti-Ro antibodies; foetal goitre; foetal hypo- or hyperthyroidism; foetal growth restriction	[5,68,69]

APLA—antiphospholipid antibodies; APS—antiphospholipid syndrome; LA—Lupus Anticoagulant; PE—Pre-eclampsia.

**Table 6:** Routine evaluation of a SLE patient preparing for pregnancy.

Complete clinical evaluation (anamnesis and physical exam) to observe for active disease or contraindication
Lab evaluation: complete blood count, proteinuria, urinalysis, renal and hepatic function, glucose level, coagulation tests, inflammatory markers, 25(OH)D in the presence of risk factor
Immune markers associated with active disease: low complement level (C3, C4, C1q) and increased anti dsDNA
Autoantibodies associated with maternal or foetal complications: APLA, anti-Ro, anti La antibodies, antithyroid antibodies
Collect scores to quantify activity: SLEDAI-2K and LLDAS
Check for existence of damage that might contraindicate pregnancy: pulmonary hypertension, low pulmonary function, cardiac failure, severe kidney failure, stroke, or major thrombosis
Check specific disease medications
Check comorbidities medication (antihypertensive), LDA, Vitamin D, Folic Acid
Check approved vaccination status

APLA—antiphospholipid antibodies; anti dsDNA- anti-double-strand DNA; C—Complement; 25(OH)D—25-hydroxy-Vitamina D; LDA—Low-Dose Aspirin; LLDAS—Lupus Low Disease Activity State; PE—Pre-Eclampsia; SLEDAI-2K—Systemic Lupus Erythematosus Disease Activity Index 2000.

**Table 7:** Importance of pre-conception evaluation: when to defer or to contraindicate an SLE pregnancy.

Evaluation	Deferred Conception	Contraindication for Pregnancy
Active disease in the last 6 months	✓	
Active lupus nephritis (still need for teratogenic regimen)	✓	
Severe pulmonary hypertension (>50 mmHg)		✓
Severe restrictive lung disease (forced vital capacity < 1 L)		✓
Advanced renal insufficiency (creatinine > 2.8 mg/dL)		✓
Advanced heart failure		✓
Stroke or major thrombotic event in the last 6 months	✓	
Previous PE or HELLP (Homolysis, Elevated Liver enzyme and Low Plates) syndrome despite correct treatment		✓
Forbidden medication: changed first to approved medication and weight a few months to observe	✓	
Vaccination status: missing recommended vaccines	✓	

PE—Pre-eclampsia.

## Data Availability

Not applicable.
